# Determinants of orphan drug health technology assessment in South Korea: an empirical analysis

**DOI:** 10.3389/fphar.2025.1619984

**Published:** 2025-09-02

**Authors:** Se Hee Lee, Sung Kyung Lee, Jong Hyuk Lee, Joon Seok Bang

**Affiliations:** ^1^ College of Pharmacy, Sookmyung Women’s University, Seoul, Republic of Korea; ^2^ Korea Institute for Industrial Economics and Trade, Sejong, Republic of Korea; ^3^ College of Pharmacy, Chung-Ang University, Seoul, Republic of Korea

**Keywords:** orphan drug, rare diseases, pricing, health technology assessment, reimbursement, budget impact, external reference pricing

## Abstract

**Background:**

Orphan drug pricing in South Korea, including its workings and determinants, is a complicated aspect of the country’s healthcare system. Therefore, this study identified the factors associated with the pricing of orphan drugs in South Korea.

**Methods:**

The study screened 66 orphan drugs out of the whole set of newly reimbursed drugs from 2012 to 2022. The analysis presented the characteristics of the orphan drugs and accounted for correlations between treatment cost and other seemingly related variables. Formal multivariate regression analysis indicated associated factors in multivariate settings.

**Results:**

Annual treatment cost per patient displayed a weakly negative correlation with the number of patients. Meanwhile, treatment cost exhibited a strongly positive correlation with the average of adjusted prices in seven advanced countries, reaching an adjusted R-squared value of 0.99. Treatment cost also showed a positive correlation with budget impact.

**Conclusion:**

The pricing of orphan drugs in South Korea is predominantly influenced by external reference pricing. Drugs targeting smaller patient populations tend to be priced higher. This finding can guide policymakers, pharmaceutical companies, and healthcare providers in their efforts to balance patient accessibility with the sustainable development of orphan drugs and the financial sustainability of public health insurance.

## 1 Introduction

With advances in medical and diagnostic technologies, as many as 6,000–8,000 rare diseases have been identified, and so the number of patients diagnosed with rare diseases has been on the rise (Boycott and Ardigó, 2018; Nguengang Wakap et al., 2020; Baynam et al., 2024). A significant proportion of rare diseases are chronic, progressive, debilitating, life-threatening, and/or fatal. As such, unmet medical needs with respect to rare diseases have led to strategic investments in these therapeutic areas (Dunoyer, 2011; Rodriguez-Monguio et al., 2017; Boycott and Ardigó, 2018). However, rare diseases tend to have extremely low prevalence, with some occurring in a mere 1–5 per 1,000,000 people (Nguengang Wakap et al., 2020). The poor prospect of commercial success, given the smaller-sized patient pool, has worked against the strategic business planning of profit-oriented pharmaceutical companies in this area (Gammie et al., 2015).

However, patients with rare diseases collectively account for 4%–8% of the general population (Nguengang Wakap et al., 2020; Baynam et al., 2024). Moreover, disease impact reverberates throughout the lives and livelihoods of patients and their families. As many as 50% of those affected are children, 30% of whom will die before they reach the age of 5 years (Gammie et al., 2015; The Lancet Diabetes, 2019; Chan et al., 2020; Nguengang Wakap et al., 2020).

After the US introduced the *Orphan Drug Act* in 1983, other developed countries, including the EU and Japan, have followed suit. Relevant initiatives have included the designation of orphan drugs (ODs), marketing approval and market exclusivity, and a raft of financial incentive programs particularly earmarked for rare diseases (Gammie et al., 2015; Chan et al., 2020). Presently, over half of all countries provide subsidies for the research and development (R&D) of ODs (Braun et al., 2010; Gammie et al., 2015; Chan et al., 2020; Postma et al., 2022). Subsequent market interest has led to the filing of as many as 5,099 OD applications in the US; as of 2019, 724 pipelines (14%) have successfully reached the final approval stage (Miller et al., 2021). Incentive legislation for orphan drugs has thus effectively contributed to their development, thereby addressing the unmet needs of patients with rare diseases (Michaeli et al., 2023; Ipe et al., 2024; Martins et al., 2024).

The sheer scale of R&D investment that has poured into the OD pipelines has naturally translated into exorbitant pricing for such drugs, in turn limiting patient access. As such, the price setting of ODs is intertwined with issues of patient accessibility and commercial viability for pharmaceutical companies. Despite the intervention of state-led initiatives, a wide range of discrepancies persist in terms of drug accessibility, with the large bulk of approved ODs remaining uninsured and stuck in reimbursement negotiation. Only approximately 65% of ODs approved in the EU are publicly insured as of 2018; in South Korea, the proportion was 56% in 2019 (Stawowczyk et al., 2019; Lee et al., 2020).

Reimbursement approvals are directly linked to patient accessibility—ODs would not be financially feasible options to most patients without reimbursements (Stawowczyk et al., 2019). However, pricing & reimbursement (P&R) decisions require much caution as the number of ODs entering markets continue to rise and budgetary burdens become heavy (Hughes-Wilson et al., 2012; Hall and Carlson, 2014; Logviss et al., 2016; Czech et al., 2019). The pricing levels of ODs directly translate to pharmaceutical companies’ return on investment and, ultimately, long-term financial viability (Denis et al., 2010; Côté and Keating, 2012; Franco, 2013; Berdud et al., 2020). Therefore, the pricing of ODs has great importance to all three parties involved—patients, pharmaceutical companies, and policymakers.

Many countries have no specialized P&R pathways that apply only to ODs, opting instead for flexibilities and exceptions in their pricing systems, such as the use of conditional incremental cost-effectiveness ratio (ICER) threshold, weighted quality-adjusted life year (QALY), pharmaco-economic evaluation (PE) waivers, and risk-sharing agreements (RSAs) (Gammie et al., 2015; Felippini et al., 2024). Some countries have specialized funds that are earmarked for expensive medicines for fatal and rare diseases (Gammie et al., 2015; Felippini et al., 2024). To consider the incorporation of methods for assessing societal values and preferences within health technology assessments for ODs, P&R models such as multi-criteria decision analysis, proportional time trade-off, and discrete choice experiments are also examined (de Andrés-Nogales et al., 2021; Vásquez et al., 2024).

Four P&R pathways apply to newly approved ODs in South Korea: essential ODs, PE-waived ODs, weighted average price (WAP) ODs, and PE ODs. For serious and life-threatening rare diseases, RSAs can be applied (Supplementary Figure S1) (Lee, 2021). Essential and PE-waived ODs should meet a set of prescribed criteria and can be listed based on the adjusted pricing of seven advanced countries (A7 countries: the US, United Kingdom (UK), Germany, France, Italy, Switzerland, and Japan) without proving cost-effectiveness (Supplementary Table S1) (Bang and Lee, 2021). The pricing of WAP ODs is computed based on the WAP of alternatives. PE ODs can be listed up to premium prices with the support of PE studies. External reference pricing (ERP) refers to the average of adjusted A7 prices; the listing prices of newly approved drugs should not exceed average levels. Especially for PE-waived new drugs, the lowest value of adjusted A7 prices should be used as the reference price. Adjusted prices start from ex-factory rates, upon which exchange rates, value added tax, and distribution margins are applied and added (Kim et al., 2021).

In this study, we attempt to determine the factors associated with the pricing of ODs in South Korea with the goal of elucidating the workings and determinants of OD pricing there.

## 2 Materials and methods

### 2.1 Data sources

**FIGURE 1:**
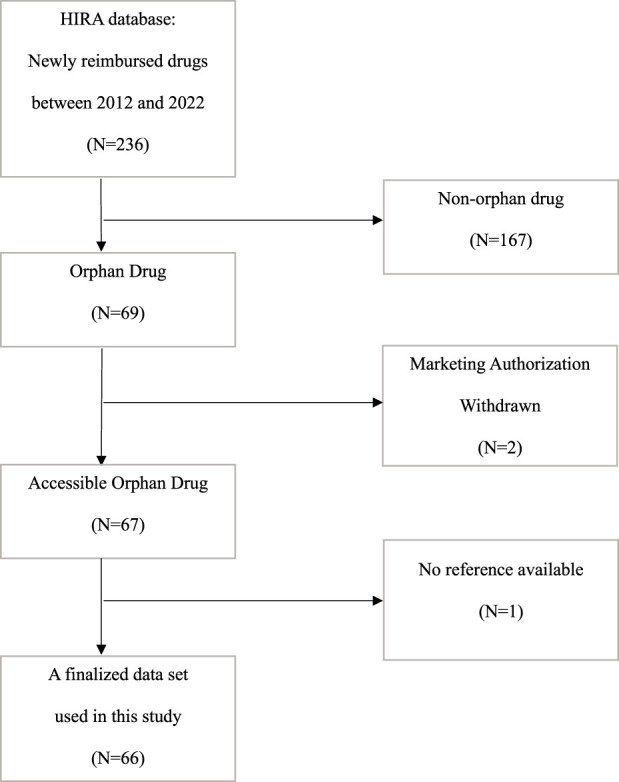
Flowchart for data screening HIRA, health insurance review and assessment service; OD, orphan drug; MA, marketing authorization.

We screened 66 ODs out of the whole set of newly reimbursed drugs from 2012 to 2022 from the official website of the Health Insurance Review & Assessment Service (HIRA) (Figure 1) (HIRA, 2024b). We picked out patient numbers, anatomical therapeutic chemical classification system (ATC), number of listed countries among the A7, modality, and the average of A7 adjusted prices as potential explanatory variables (Table 1). The respective data on each variable were obtained from HIRA Drug Reimbursement and Evaluation Committee reports (HIRA, 2024a). The A7-adjusted prices were calculated from official drug pricing sources of the respective A7 countries as available prior to the reimbursement decision in South Korea for each product. These sources included the Redbook Index (US), Monthly Index of Medical Specialities (UK), ROTE Liste (Germany), VIDAL (France), L'Informatore Farmaceutico (Italy), Arzneimittel Kompendium (Switzerland), and Hokenyaku Jiten published by Yakugyo Kenkyukai (Japan) (HIRA, 2025). In line with previous research, we used annualized treatment costs to standardize OD prices using the information found in the summary of product characteristics and Korean National Health Insurance Service reimbursement conditions (Jommi et al., 2021; Villa et al., 2022). If the recommended dosage exceeded one, we used the annualized price for multiple shots; if recommended dosage was only one for a full year, we used the price for a single administration. All cost data were converted from Korean won (KRW) to US dollars (USD) using the 2022 average annual exchange rate (1 USD = 1,294 KRW) based on data from the Bank of Korea.

**TABLE 1:** Description of variables included in the analysis.

Variables	Data type	Definition
Dependent variable		
Annual treatment cost per patient	Continuous	Calculated treatment cost for standardizing orphan drug prices using the information found in the summary of product characteristics and NHIS reimbursement conditions.
Independent variable		
A. Rarity		
Number of patients	Continuous	Estimated number of prevalent patients with target disease condition.
B. Budget impact		
Estimated budget impact per year	Continuous	Estimated total medication cost per year if all target patients were treated with the drug.
C. Listing status of reference countries		
Average of A7 adjusted prices (USD)^1^	Continuous	Average adjusted listing prices of the drug in A7 countries.
Number of listed countries among A7	Continuous	Number of countries already listed among reference A7 countries.
Rate vs. average of A7 adjusted price	Continuous	Ratio of listing price in South Korea compared with average of A7 adjusted prices.
D. P&R pathways		
Weighted average price (WAP)	Categorical (Yes/No)	Pricing based on alternatives’ weighted average price.
Pharmaco-economic evaluation (PE)	Categorical (Yes/No)	Pricing based on PE.
PE waiver	Categorical (Yes/No)	Pricing based on average A7 adjusted prices without PE.
RSA assignment	Categorical (Yes/No)	Risk sharing agreement application.
E. Indication		
Onco drug category	Categorical (Onco/Non-Onco)	Whether the drug is for rare-oncology treatment.
Anatomical therapeutic chemical (ATC) classification (1st Level)	Categorical	Anatomical or pharmacological groups of the drug’s active substances.

A7: Advanced seven countries (US, UK, Germany, France, Italy, Switzerland, and Japan; P&R, pricing and reimbursement).

### 2.2 Analysis methods

We used descriptive analysis to present the characteristics of the ODs. We applied Fisher’s exact test on the differences of RSA assignment patterns between oncology and non-oncology drugs. We broke down the annual treatment cost per patient, our dependent variable, by P&R pathways, RSA assignment, and the anatomical therapeutic chemical (ATC) classification system. We then looked into correlations between treatment cost and other seemingly related variables. We also conducted formal multivariate regression analyses to identify associated factors in multivariate settings. We used R version 4.3.1 to carry out statistical analysis.

Prior to conducting analysis, we found that both the distributions of annual treatment cost per patient in South Korea—our variable of interest—and the corresponding A7 adjusted average prices—one of our explanatory variables—displayed highly skewed patterns to the left, with a few extremely highly priced ones set apart as outliers (Supplementary Figure S2). Hence, we used *log*-transformed versions of both variables to make them exhibit a normalized distribution in our regressions.

## 3 Results

### 3.1 Descriptive analyses

Table 2 presents a summary of the results of the descriptive analyses. Among the distinctive characteristics, we found that ODs typically received a PE waiver, as found in 26 of the 66 ODs (39.4%), or WAP, noted in 21 ODs (31.8%). Less than a third (19, 28.8%) passed through PE. ODs could be roughly evenly classified into oncology (32, 48.5%) and non-oncology indications (34, 51.5%). The results of Fisher’s exact test showed that all differences were significant, except for the PE-waived case (Table 3). PE-waived ODs were the most likely to receive RSA assignment compared with other pathways, regardless of oncology indication. No non-oncology drugs that passed through WAP were RSA applied.

**TABLE 2:** Summary of descriptive analyses.

Variables (*N =* 66)				Statistics
Continuous variables	Annual treatment cost per patient (USD)			97348.5 (±216180.4)
	Average of A7 adjusted prices (USD)			1288.8 (±2553.3)
	Number of patients			519.4 (±1116.8)
	Budget impact/year (USD)			6722323.8 (±9007838.3)
	Number of listed countries among A7			5.2 (±1.5)
Categorical variables	Oncology (N)		Yes	32 (48.5%)
			No	34 (51.5%)
	P&R pathway (N)	WAP	Yes	21 (31.8%)
			No	45 (68.2%)
		PE	Yes	19 (28.8%)
			No	47 (71.2%)
		PE waiver	Yes	26 (39.4%)
			No	40 (60.6%)
	RSA (N)		Yes	36 (54.5%)
			No	30 (45.5%)
	ATC (N)	A (alimentary tract and metabolism)		8 (12.1%)
		B (blood and blood forming organs)		5 (7.6%)
		C (cardiovascular system)		1 (1.5%)
		J (general anti-infective systemic)		5 (7.6%)
		L (antineoplastic and immunomodulating agents)		42 (63.6%)
		M (musculoskeletal system)		2 (3.0%)
		N (nervous system)		2 (3.0%)
		V (various)		1 (1.5%)

Note: descriptive statistics tabulate means and standard deviations for continuous variables and frequencies and percentages for categorical variables.

A7, US, UK, Germany, France, Italy, Switzerland, Japan; P&R, pricing and reimbursement; WAP, weighted average price; PE, pharmaco-economic evaluation; RSA, risk sharing agreement; ATC, anatomical therapeutic chemical classification system.

**TABLE 3:** Breakdown of orphan drugs by the pricing and reimbursement pathway, oncology indications, and risk sharing agreement assignment.

P&R pathway	Oncology (RSA applied)	Non-oncology (RSA applied)	p-value	Total
WAP	5 (3, 60%)	16 (0, 0%)	0.00	21 (32%)
PE	11 (8, 73%)	8 (2, 25%)	0.03	19 (29%)
PE waiver	16 (14, 88%)	10 (9, 90%)	0.84	26 (39%)
Total	32 (25, 78%)	34 (11, 32%)	0.00	66

Note: Fisher’s exact test was used to test differences in proportions when null claim was that the proportions were the same.

P&R, pricing and reimbursement; WAP, weighted average price; PE, pharmaco-economic evaluation; RSA, risk sharing agreement.

**FIGURE 2:**
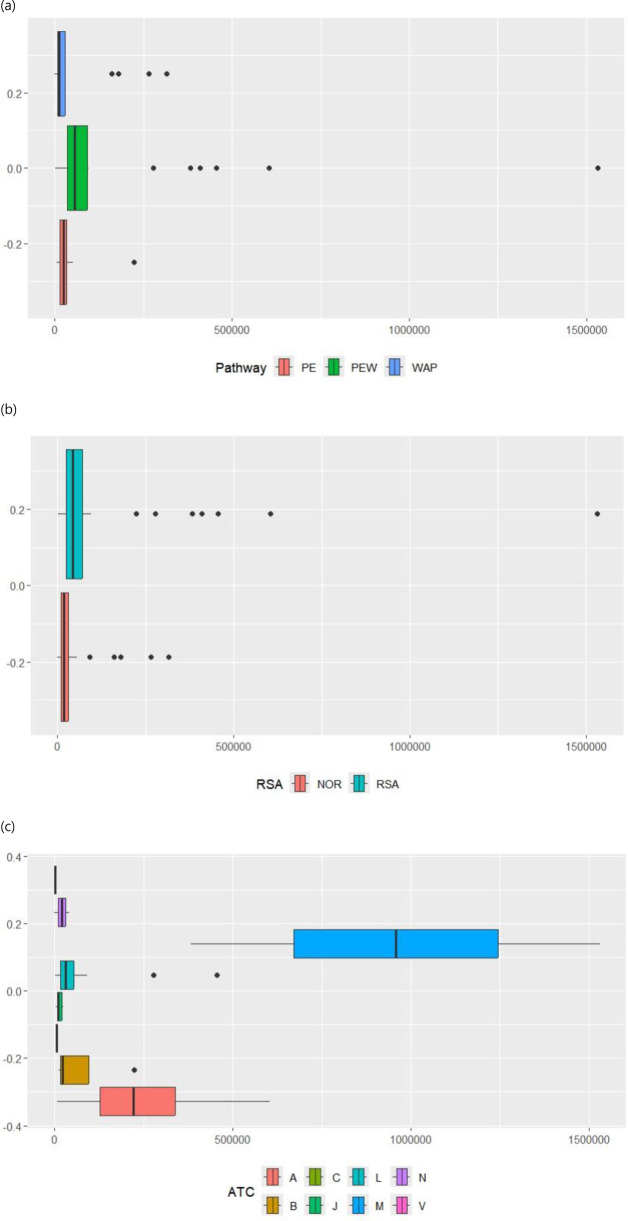
Box plots of annual treatment cost per patient by pricing and reimbursement pathway, risk-sharing agreement, and anatomical therapeutic chemical classification system. **(a)** Annual treatments per patient using the pricing and reimbursement pathway. **(b)** Annual treatment per patient with risk-sharing agreement. **(c)** Annual treatment per patient by anatomical therapeutic chemical classification system. X-axis represents annual treatment cost per patient in USD (exchange = 1,294 KRW/USD, average annual exchange rate of 2022 based on data from the Bank of Korea).

On average, the PE-waived ODs tended to have much higher average treatment costs, USD 178,006—almost double the average of all ODs at USD 97,349 and quadruple the average of the not-PE-waived drugs. This variation may be attributed to the extreme outlier cases, mostly in the PE waiver pathway (Table 4; Figure 2a). Similarly, the RSA-assigned drugs had higher average treatment costs than the not-RSA-assigned cases; the median gap tended to be much smaller (Table 4; Figure 2b). Meanwhile, other drugs that passed through either PE or WAP had lower averages than non-PE and non-WAP cases.

**TABLE 4:** Average annual treatment cost per patient by the pricing and reimbursement pathway and anatomical therapeutic chemical classification system.

Variables			Mean (STD)	Median	Range (Min, Max)
P&R pathway	WAP	N (*N =* 45, 68%)	117,586 (252,725)	42,139	(1,815, 1,531,474)
		Y (*N =* 21, 32%)	53,982 (92,064)	11,333	(34, 315,153)
	PE	N (*N =* 47, 71%)	122,591 (250,808)	40,026	(34, 1,531,474)
		Y (*N =* 19, 29%)	34,905 (47,184)	23,598	(4,714, 222,288)
	PE waiver	N (*N =* 40, 61%)	44,921 (73,940)	21,619	(34, 315,153)
		Y (*N =* 26, 39%)	178,006 (319,086)	58,987	(1,815, 1,531,474)
RSA	N (*N =* 30, 45%)		47,353 (78,369)	17,551	(34, 315,153)
	Y (*N =* 36, 55%)		139,011 (278,882)	43,220	(1,667, 1,531,474)
ATC	Alimentary tract and metabolism (A) (*N =* 8)		245,916 (198,762)	221,456	(6,799, 604,187)
	Blood and blood forming organs (B) (*N =* 5)		73,186 (90,186)	21,596	(11,243, 222,288)
	Cardiovascular system (C) (*N =* 1)		4,714	4,714	N.A.
	Anti-infectives for systemic use (J) (*N =* 5)		13,230 (8,310)	10,238	(4,161, 23,254)
	Antineoplastic and immunomodulating agents (L) (*N =* 42)		49,151 (77,622)	30,490	(1,667, 455,041)
	Musculo-skeletal system (M) (*N =* 2)		957,326 (811,967)	957,326	(383,179, 1,531,474)
	Nervous system (N) (*N =* 2)		20,030 (28,279)	20,030	(34, 40,026)
	Various (V) (*N =* 1)		1,815	1,815	N.A.
Total (*N =* 66)			97,349 (216,180)	29,066	(34, 1,531,474)

Note: units are USD (exchange = 1,294 KRW/USD, average annual exchange rate of 2022 based on data from the Bank of Korea).

P&R, pricing and reimbursement; WAP, weighted average price; PE, pharmaco-economic evaluation; RSA, risk sharing agreement; ATC, anatomical therapeutic chemical classification system.

A breakdown by ATC classification indicated that a majority of ODs (42 of 66, 63.6%) fell under code L, representing antineoplastic and immunomodulating agents. The average cost of L-coded ODs, USD 49,151, was almost half that of the entire dataset of USD 97,348, but its median was slightly higher than the median of the entire set (Table 4; Figure 2c). The mean and median of ODs classified as musculoskeletal system (M), which had only two observations, were ten and three times higher, respectively, than those of the entire dataset.

### 3.2 Regression analysis

**FIGURE 3:**
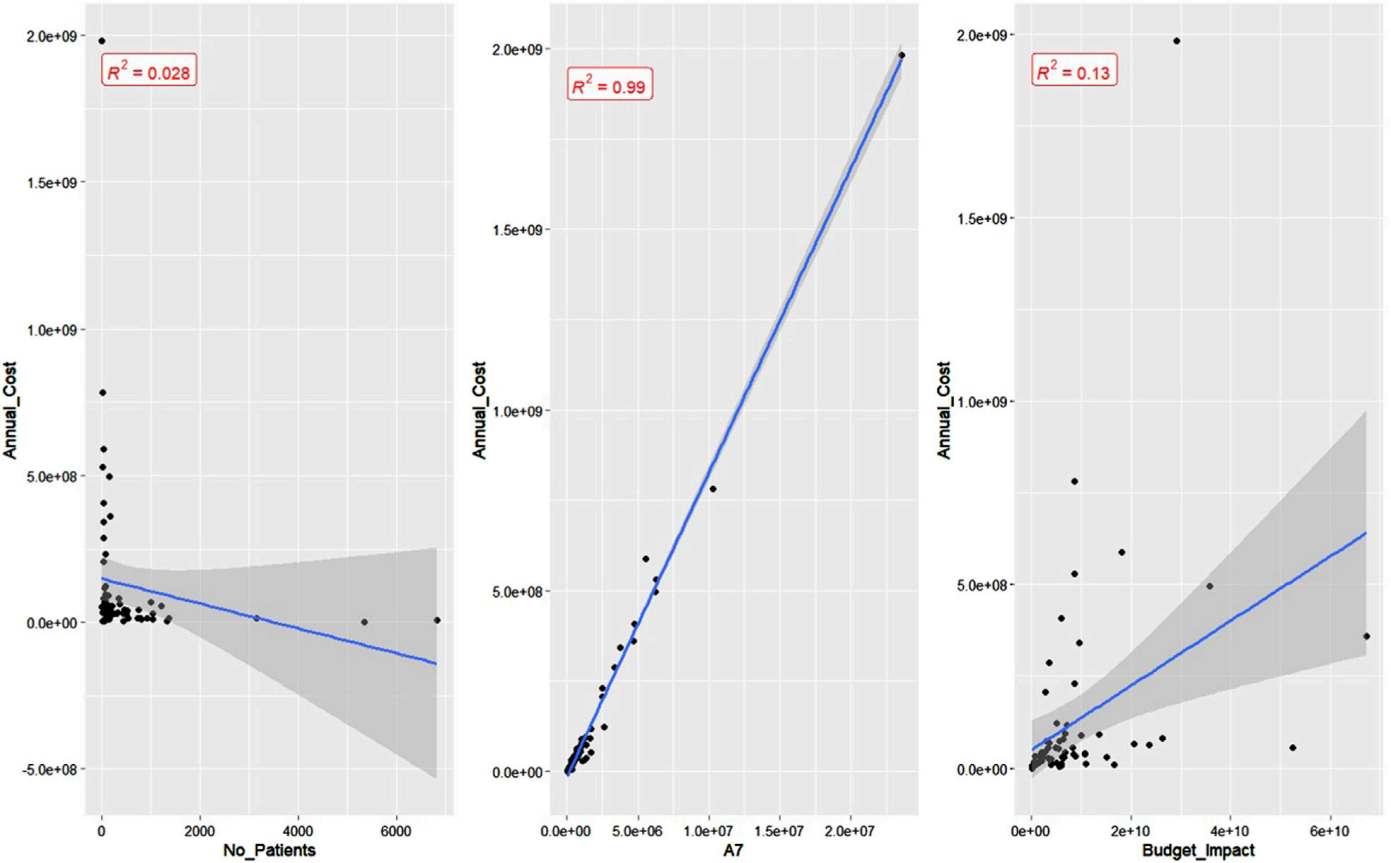
Scatterplots of annual treatment cost per patient against major variables. 1. Number of patients. 2. Average adjusted prices of advanced seven countries (US, UK, Germany, France, Italy, Switzerland, and Japan). 3. Budget impact.

Before conducting regression analysis, we drew scatterplots between the average annual treatment cost per patient in South Korea, which is our dependent variable of interest, and three seemingly associated variables: number of patients, average of A7 adjusted prices, and budget impact (Figure 3). The annual treatment cost per patient showed a weakly negative correlation with the number of patients, a strongly positive correlation with the average of adjusted A7 prices (reaching an adjusted *R*-squared value of 0.99), and a positive correlation with budget impact. The scatterplots of *log*-transformed variables are presented in Supplementary Figure S3.

Regarding the regression analysis, we set the *log*-transformed annual treatment cost per patient as our dependent variable and regressed it on dummy variables for oncology indication, ATC classification, P&R pathway, and RSA assignment, and continuous variables for number of patients, budget impact, number of listed countries among A7 countries, and *log*-transformed average of adjusted prices in A7 countries (Table 5). To avoid multicollinearity, we checked the variance inflation factor (VIF) scores. For both the regression equations of a full specification and a model without the ATC classification dummy variables, we noted that both F-statistics and adjusted R-squared values displayed fairly good signs of model fitness.

**TABLE 5:** Multivariate linear regression results for annual treatment costs per patient.

Regressor		*Log*(Annual treatment cost per patient) (1)	*Log*(Annual treatment cost per patient) (2)
(Intercept)		1.55 (0.000***)	1.276 (0.000***)
Oncology	Oncology	−0.148 (0.011*)	−0.084 (0.122)
	Non-oncology (reference variable)		
ATC	A	Reference variable	-
	B	−0.224 (0.007*)	
	C	−0.218 (0.171)	
	J	0.104 (0.253)	
	L	−0.062 (0.382)	
	M	−0.167 (0.158)	
	N	−0.695 (0.000***)	
	V	−0.184 (0.274)	
P&R pathway	PE (reference variable)		
	PE Waiver	−0.003 (0.952)	−0.033 (0.600)
	WAP	−0.072 (0.152)	−0.067 (0.303)
RSA	RSA	0.158 (0.002**)	0.104 (0.102)
	Non-RSA (reference variable)		
Number of patients		−0.00005 (0.013*)	−0.00006 (0.015*)
Budget impact		0.000 (0.453)	0.000 (0.493)
Number of listed countries among A7		0.022 (0.066*)	0.024 (0.134)
*Log*(average of adjusted prices in A7)		1.035 (0.000***)	1.068 (0.000***)
*F* statistic		121.1 (0.000)	116.5 (0.000)
Adjusted *R*-squared ( $\hat{R}$ ^2^)		0.965	0.934

Note: Inputs are estimated beta coefficients ( $\hat{\beta }$ ) and numbers in parentheses are p-values. *** denotes corresponding coefficient is statistically significant at a significance level (α) of 1%, ** for the case of α = 5%, and * for the case of α = 10%. Since the dependent variable and one of the independent variables are log-transformed, coefficients for log-transformed independent variables are interpreted as the expected percentage change in y in response to a 1% change in $x_{j}$ and coefficients for other regressors as expected (100 * $\hat{\beta_{j}}$ ) % for a unit increase in $x_{j}$ .

P&R, pricing and reimbursement; WAP, weighted average price; PE, pharmaco-economic evaluation; RSA, risk sharing agreement; ATC, anatomical therapeutic chemical classification system; A7, US, UK, Germany, France, Italy, Switzerland, and Japan.

Regarding the significance of individual variables, both regression equations showed that the number of patients was negatively associated with *log*-transformed average treatment cost. In other words, the *log* of the average treatment cost could be expected to decrease by 0.005%, −0.005% = (100* $\hat{\beta_{j}}$ )% = (100* – 0.00005)%, for the full specification and by 0.006% for the model without the ATC classification in response to each additional patient increase. For both regression models, a 1% change in *log*-transformed adjusted average prices in the A7 was associated with a $\beta_{j}$ % change in *log*-transformed average treatment cost. As such, a 1% change in *log*(average of A7 adjusted prices) was estimated to correspond to a 1.035% increase in *log*(average treatment cost) for the full model and a 1.068% increase for the partial model.

In the full model with a higher adjusted-*R* value and *F*-statistic, a classification as an oncology drug was associated with a −14.8% change in *log*(average treatment cost) compared with non-oncology cases. B- and N-coded drugs (for blood and blood-forming organs and for nervous system, respectively) in the ATC classification were associated with changes of −22.4% and −69.5% in *log*(average treatment cost), respectively. Meanwhile, RSA-assigned drugs were associated with a 15.8% change in *log*(average treatment cost).

Both scatterplots and regression results indicated an almost one-to-one correspondence between average treatment cost in South Korea and adjusted average prices in A7 countries, indicating that the OD pricing in South Korea was highly influenced by the changes in adjusted average prices in A7 countries. The intrinsic characteristics of drugs, such as oncology indication and ATC classification, and RSA assignment status also had much influence. Lastly, the number of patients made a small degree of difference. Meanwhile, differences in P&R pathways and budget impact do not incur changes in average treatment cost, contrary to our expectation.

Therefore, the drug characteristics that qualified ODs to certain P&R pathways and other external factors collectively characterized the pricing of ODs. Notably, the pricing of ODs in South Korea did not move in tandem over different P&R categories. The P&R pathway dummy variables collectively contributed to the overall model fitness with other explanatory variables. Meanwhile, the strong statistical association with adjusted average prices in A7 countries indicated that reference prices had the greatest impact on the pricing of ODs in South Korea.

## 4 Discussion

P&R pathways have been widely regarded as the most important factor determining patients’ access to ODs (Gammie et al., 2015). Given that ODs are used by only a small fraction of the whole population, most are priced at an astronomical level to compensate for their R&D expenditure (Michaeli et al., 2023). Therefore, patients who need ODs face difficulties in affording such prices without the help of public reimbursement (Stawowczyk et al., 2019). For biopharmaceutical companies, having their drugs listed in the reimbursement system encourages consumption, which can help them recoup their hefty investment expenses in a financially viable and long-term way . Otherwise, even companies with pipelines whose safety and efficacy have passed through formal evaluation processes can go bankrupt if cashflow shortfalls occur (Rollet et al., 2013; Stawowczyk et al., 2019).

As more ODs enter the market, P&R decisions are becoming a major budgetary issue with which policymakers must contend. In South Korea, the expenditure share of ODs out of total pharmaceutical spending nationwide accounts for a mere 1%, below the 3% level of most European countries, and in the US or Bulgaria it can reach 5% or even to 9% (Teagarden et al., 2014; Gombocz and Vogler, 2020). Ultimately, decisions boil down to a moral dilemma between *utilitarianism*—maximizing outcomes with finite healthcare resources—and *non-abandonment*, which can be seen at favoring those in dire need (Onakpoya et al., 2015; Kanavos et al., 2020; Zimmermann et al., 2021; Postma et al., 2022).

The annual treatment costs per patient of existing ODs range between USD 1,300 and 500,000, with the average cost per patient between USD 32,000 and 35,000 (Schey et al., 2011; IQVIA, 2020). Meanwhile, the return on investment of ODs at 8.4% far exceeds that of non-orphan drugs at 2.3%—a fact that raises suspicion that profit-oriented corporates tap patients’ willingness to pay in the absence of alternative treatments (Rzakhanov, 2008; Côté and Keating, 2012; Berdud et al., 2020). In 2008, 43 blockbusters drugs, or drugs with sales exceeding USD 1 billion, had orphan designations (Côté and Keating, 2012).

All ODs that are included in this study were developed by foreign companies and launched first in other countries and then entered the South Korean market. Since the Korean government runs the ERP system, it resulted in almost a one-to-one correspondence between adjusted A7 and Korean OD prices, leaving only limited scope for the influence of other determinants. Given that most advanced countries implement variants of the ERP system, once-determined pricings inevitably spill over to other neighboring countries and, thus, prices worldwide are aligned in some sense. Accordingly, pharmaceutical companies exploit the situation by delaying new drug launches in countries where they are highly likely to be compelled to list low prices and withdrawing marketing approvals upon unsatisfactory negotiations in the P&R review processes (Rollet et al., 2013; Kanavos et al., 2020).

In terms of regulatory harmonization, South Korea is placed ahead of the US, EU, and UK, but in terms of flexibility it is placed behind China (IQVIA, 2024). South Korean authorities have introduced layers of the abovementioned pathways in an attempt to accommodate accessibility for patients in dire need. Among the available P&R pathways in South Korea, ODs are given PE waivers the most (39%), then WAP (32%) and PE (29%). Drugs that go through the PE waiver pathway are also the most likely to be assigned an RSA compared to the other P&R pathways. Notably, orphan drugs that passed through PE waiver and are assigned an RSA tend to have higher average annual treatment costs than those that go through WAP or PE pathways.

Our study indicates that neither the difference in P&R pathways nor the total expected budget of an individual OD significantly influences its pricing. However, we found a strongly positive correlation with adjusted A7 prices—in other words, the higher the adjusted A7 price, the higher the corresponding price in South Korea. Instead, the intrinsic characteristics of drugs, such as oncology status, ATC classification, and RSA assignment status, play a more substantial role in determining prices. While the expected budget of an individual OD does not appear to be statistically associated with individual OD pricing, it remains unclear whether this reflects an intentional exclusion of fiscal considerations or limitations in how such impact is operationalized in the pricing decision process.

Our findings suggest that while P&R pathways are structurally diverse and multi-layered in South Korea, pricing decisions are consistently driven by objective metrics such as A7 prices and drug characteristics. This indicates that a transparent and rule-based pricing environment is functioning there. In light of the global trend of increasing OD approvals and their substantial budgetary impact, South Korea’s approach may serve as a reference for countries seeking balance between patient accessibility and fiscal sustainability in OD policy.

Our study has several limitations. First, in South Korea, RSAs, which require mandatory pay-back if sales exceed prespecified thresholds, represent one of the most financially supportive mechanisms for payers. As such, the listed and adjusted prices may have unrealized gaps and may not match the actual prices paid by payers and patients. In our study, as many as 54.5% of ODs included (i.e., 36 drugs) were classified as RSAs. However, we chose not to apply a sensitivity analysis to adjust for RSA refund, as similar refund-type RSAs are also common in comparator countries. Second, we calculated the annual treatment cost per patient based on the dosage regimen approved by the Korea Ministry of Food and Drug Safety (KMFDS). However, in real-world cases, treatment costs may be influenced by clinical practice guidelines or reimbursement criteria. As such, the actual prices may differ from the prices that we calculated.

## 5 Conclusion

Our study delineates the factors having actual influence on orphan drug (OD) pricing, highlighting the interplay between OD pricing and external reference prices, drug characteristics, and the size of the treatment-eligible patient population. In light of these results, we recommend the adoption of a flexible approach to OD pricing decisions to ensure that patients with ultra-rare diseases are not marginalized in terms of treatment access. Our findings can guide policymakers, pharmaceutical companies, and healthcare providers in their efforts to balance patient accessibility with OD development and the financial sustainability of public health insurance.

## Data Availability

The original contributions presented in the study are included in the article/Supplementary Material; further inquiries can be directed to the corresponding authors.
