# Supplementary material for: Determinants of orphan drug health technology assessment in South Korea: an empirical analysis
Source: Front Pharmacol. 2025 Sep 2;16:1619984. doi: 10.3389/fphar.2025.1619984 (PMC12436107; doi:10.3389/fphar.2025.1619984)
Supplement: Supplementary file 1 [file Supplementaryfile1.docx]

**Supplementary Figure 1. Pathways for the pricing and reimbursement evaluation of new orphan drugs in the South Korean National Health Insurance Service**


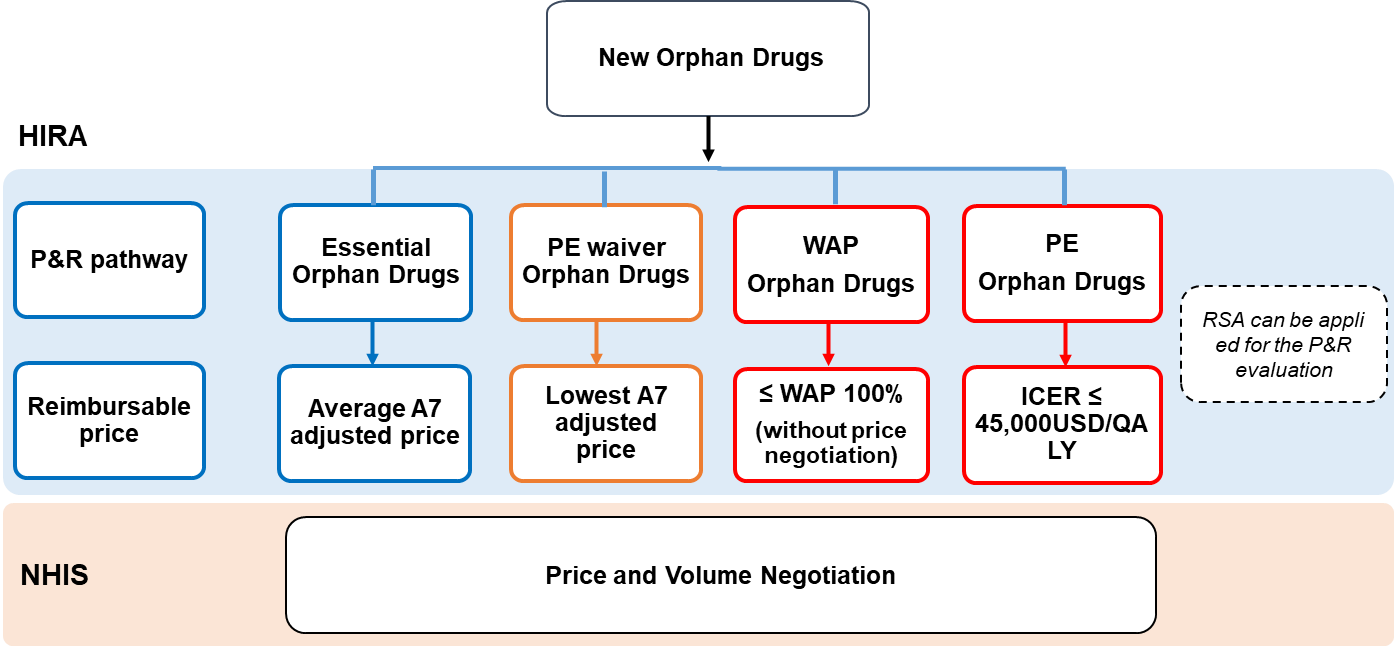


HIRA: Health Insurance Review and Assessment Service; PE: Pharmaco-economic Evaluation; WAP: Weighted Average Price; A7: US, UK, Italy, German, Japan, Swiss and France; P&R: Pricing & Reimbursement; RSA: Risk Sharing Agreement; ICER: Incrementally Cost Effectiveness Ratio; QALY: Quality-Adjusted Life Year; NHIS: National Health Insurance Service

**Supplementary Figure 2. Histogram for Annual Treatment Cost per Patient and Average of A7 adjusted prices**


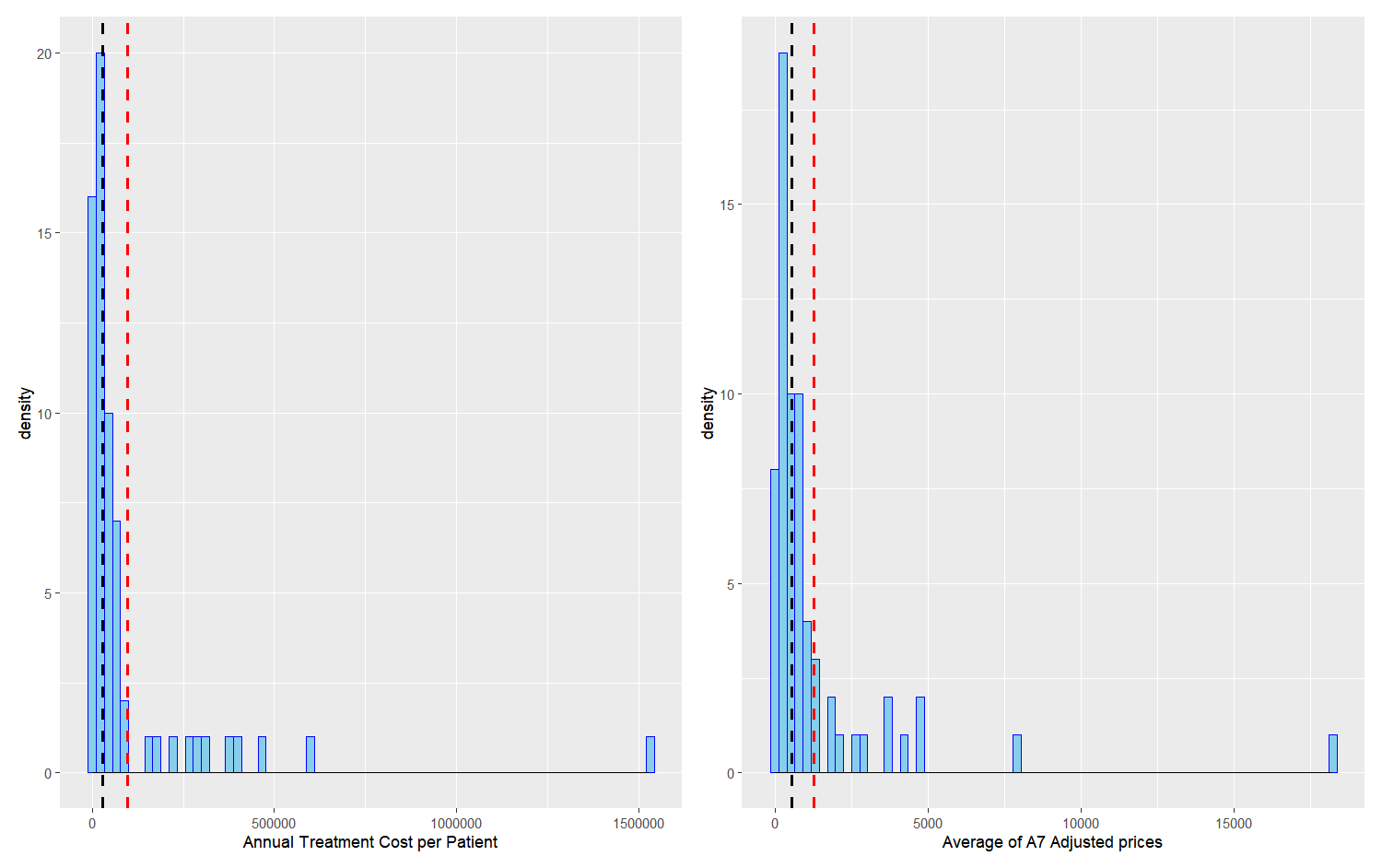


Note: The black line corresponds to median value and red line to average for our 66 observations used in this analysis. Both of treatment costs display heavily skewed distributions, with each mass concentrated around its left tail. The unit is in USD (exchange=1,294 KRW/USD, average annual exchange rate of 2022 based on data from the Bank of Korea).

**Supplementary Figure 3. log-transformed scatterplots of Annual Treatment Cost per Patient against Major Variables (1. Number of Patients, 2. Average of A7 Adjusted Prices, and 3. Budget Impact)**


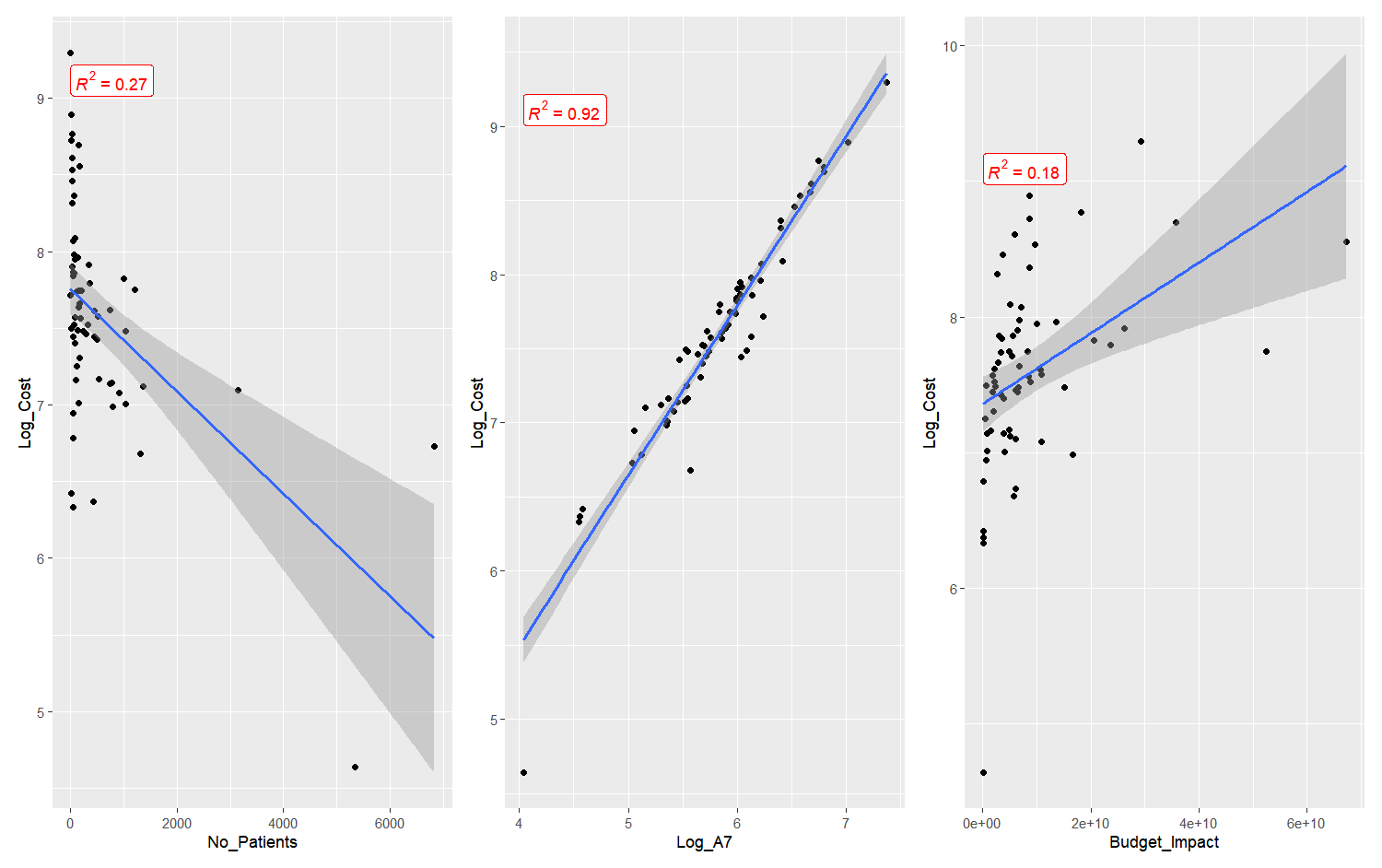


**Supplementary Table 1. Eligible criteria for the major review pathways of orphan new drug in South Korea**

| **Pathway** | **Eligible criteria** |
| --- | --- |
| Designation as essential drugs | Meet all the following criteria   1. No alternatives 2. Treating serious life-threatening conditions 3. For small patient groups (e.g., rare diseases) 4. Significant improvement in clinical efficacy or survival rate |
| PE waiver | Meet all the following criteria   1. Drugs for anticancer or rare disease treatments in case of i) no alternatives or ii) no clinical equivalent drugs or treatments, and for treating serious life-threatening conditions 2. Drugs approved with single arm clinical trial, or only based on a Phase 2 clinical trial, or difficult to generate evidence due to small number of patients 3. Listed in at least three A7 countries |
| WAP | Demonstrated clinical non-inferiority |
| PE | Demonstrated clinical superiority |

A7 countries: seven advanced reference countries (The US, The UK, Italy, German, Japan, Switzerland, and France); PE: pharmacoeconomic evaluation; WAP: weighted average price
